# Practical Notes on Diagnosis and Treatment

**Published:** 1906-08-04

**Authors:** 


### Practical Notes on Diagnosis and Treatment.

**Treatment of Lichen Planus.**

Arsenic is the drug most to be relied upon, but when, as sometimes happens, it aggravates the symptoms, antimony may be prescribed with good results. In many cases the greatest relief is obtained from biniodide of mercury.—*Mr. Malcolm Morris.*

**Adenoids and "Breathing Exercises."**

The treatment of adenoids by "breathing exercises" is entirely without value. After the lymphoid tissue has been removed it is necessary to train the patient to respire through the nose; but in themselves such exercises are utterly incapable of diminishing or removing adenoid growths.—*Sir Felix Semon.*

**Anæsthetics in Heart Disease.**

"I have seen a great deal of the administration of anæsthetics as an operator, and years ago I administered anæsthetics some thousands of times; but I have never seen a patient whose circulatory and heart condition, in the absence of pulmonary complications, was such as to be a bar to the taking of a properly administered anæsthetic."—*Sir Wm. Bennett.*

**Quinine in Corneal Ulcer.**

A solution of quinine sulphate (four grains in an ounce of water, dissolved by a trace of diluted sulphuric acid) is an excellent remedy for some cases of corneal ulcer. It should be applied by an eye-bath for five minutes four times daily. At the same time atropine is instilled and the eye bandaged. The best results are in large somewhat chronic ulcers and in marginal ulcers.

**Operations on the Thyroid Gland.**

In cases of operation on the thyroid gland reactionary fever and rapid pulse need have nothing to do with septic infection. Interference with the gland by operation may lead to these results quite independently of any septic trouble.—*Sir Wm. Bennett.*

**Febrile Periodicity in Malaria and other Diseases.**

Although tertian and quartan periodicity are absolutely pathognomonic of malaria, quotidian periodicity is of no value whatever as a diagnostic sign for or against this infection. A useful hint is sometimes got from the particular period of the day at which the individual ague-like paroxysms or rises of temperature occur. A malarial paroxysm may come on at any hour of the day or night—very often during the forenoon. The fever attending septic processes commences almost invariably in the late afternoon.—*Sir Patrick Manson.*

**Antitoxin in Diphtheritic Paralysis.**

M. Comby describes the case of a girl aged four years who suffered from diphtheritic paralysis of all four limbs and of the pharynx, and who made a complete and quick recovery after four injections of antitoxic serum repeated at intervals of three days. The paralysis occurred about four weeks after an attack of diphtheria of the throat. According to M. Barbier a dangerous and even fatal bulbar paralysis is not uncommon after diphtheria, more especially in the case of adults. The early symptoms are mental depression, anxiety, prostration, and tachycardia. The only treatment is the prompt use of a new series of injections of antitoxic serum.

